# Visual arts in the clinical clerkship: a pilot cluster-randomized, controlled trial

**DOI:** 10.1186/s12909-020-02386-w

**Published:** 2020-11-30

**Authors:** Garth W. Strohbehn, Stephanie J. K. Hoffman, Molly Tokaz, Nathan Houchens, Ruth Slavin, Suzanne Winter, Martha Quinn, David Ratz, Sanjay Saint, Vineet Chopra, Joel D. Howell

**Affiliations:** 1 Department of Internal Medicine, University of Michigan Medical School, Ann Arbor, MI USA; 2 Medicine Service, Veterans Affairs Ann Arbor Healthcare System, Ann Arbor, MI USA; 3 Division of Hospital Medicine, University of Michigan Medical School, Ann Arbor, MI USA; 4 Patient Safety Enhancement Program, University of Michigan and Veterans Affairs Ann Arbor Healthcare System, Ann Arbor, MI USA; 5 University of Michigan Museum of Art, Ann Arbor, MI USA; 6 Office of the Provost, University of Michigan, Ann Arbor, MI USA; 7 Medical Arts Program, University of Michigan Medical School, Ann Arbor, MI USA; 8 University of Michigan School of Public Health, Ann Arbor, MI USA; 9 Center for Clinical Management Research, Veterans Affairs Ann Arbor Healthcare System, Ann Arbor, MI USA; 10 Division of General Internal Medicine, University of Michigan Medical School, North Campus Research Complex, 2800 Plymouth Road, Building 16, Ann Arbor, MI 48109 USA; 11 Department of Health Management and Policy, University of Michigan, Ann Arbor, MI USA; 12 Department of History, University of Michigan, Ann Arbor, MI USA; 13 Center for Bioethics and Social Sciences in Medicine, University of Michigan, Ann Arbor, MI USA

**Keywords:** Arts in medicine, Clinical clerkship, Empathy, Grit, Mindfulness, Cluster-randomized trial

## Abstract

**Background:**

Arts exposure is associated with positive psychological constructs. To date, no randomized, controlled studies have integrated art into clinical medical education or measured its effects on positive psychological constructs or educational outcomes. In this study, we assessed the possibility and potential benefits of integrating visual arts education into a required internal medicine (IM) clinical clerkship.

**Methods:**

We conducted a controlled trial in an academic healthcare system with an affiliated art museum. IM students were assigned to one of three interventions: museum-based arts (*n* = 11), hospital-based arts (*n* = 10), or hospital-based conventional education (*n* = 13). Arts groups explored empathy, resilience, and compassion in works of art during facilitator-guided discussions. We assessed pre- and post-intervention measures of empathy, mindfulness, tolerance of ambiguity, and grit and tracked National Board of Medical Examiners IM shelf exam performance to capture changes in educational outcomes. Focus group discussions with participants in the arts-based interventions were performed at the study’s conclusion.

**Results:**

Arts education was successfully integrated into a busy clinical clerkship in both hospital and art museum settings. Focus group participants reported increased implicit bias cognizance and time for reflection, but no significant differences in psychometric or educational outcomes were identified. While most students felt positively toward the experience; some experienced distress from missed clinical time.

**Conclusions:**

This pilot study demonstrates the feasibility of integrating visual arts education into the clerkship. Although observable quantitative differences in measures of positive psychological constructs and educational outcomes were not found, qualitative assessment suggested benefits as well as the feasibility of bringing fine arts instruction into the clinical space. A larger, multi-center study is warranted.

**Supplementary Information:**

The online version contains supplementary material available at 10.1186/s12909-020-02386-w.

## Background

Medical students increasingly grapple with burnout and loss of empathy [1–6]. Once they enter clinical clerkships, learners face traumatic clinical events and an unfamiliar environment in which they perceive themselves to be constantly evaluated [7, 8]. Loss of empathy contributes to patient depersonalization and lower quality of care [9–11], while its preservation is associated with improved clinical performance reviews [12]. In prior studies, medical students who demonstrated higher empathy scores similarly obtained higher ratings of clinical competence in core clinical rotations [12].

Incorporating the visual arts into medical training may help address this struggle. One study in which 739 medical students were surveyed found that exposure to the humanities (e.g. visual arts, music, theater, literature) was significantly correlated with positive qualities, including empathy, tolerance for ambiguity, and emotional intelligence, that portend strong clinical practice skills in the future [13]. Notably, arts exposure was also inversely correlated with components of burnout, suggesting the arts improve sustainability of one’s clinical practice [13]. Operating within this paradigm, educators have tried to mitigate burnout and loss of empathy using arts-based curricular strategies [14–22]. However, these prospectively conducted studies do not offer conclusive evidence of the arts’ benefits due to their designs. Many offer opportunities only for self-selected students, others occur outside of core clinical rotations, the crucible of clinical training that is central to medical student development, and none include randomization or appropriate control groups. Prior studies have also not assessed whether introducing time for the arts will alter academic performance, an area of interest to both learners and medical schools. Moreover, many interventions are based on visits to art museums, an opportunity not readily available at all institutions. Rigorously evaluating the role of the arts in medical education would require a randomized, controlled-trial, but it is unclear whether this trial would be feasible.

In this context, we created a pilot study aimed to assess the possibility and potential benefits and drawbacks of integrating the visual arts into an intense, graded, core internal medicine (IM) clinical clerkship. The pilot study utilizes a systematic study design and incorporation of relevant population and treatment groups with both quantitative and qualitative outcome measures toward an objective of informing a larger, multi-center study.

## Methods

### Research design

**Fig. 1:**
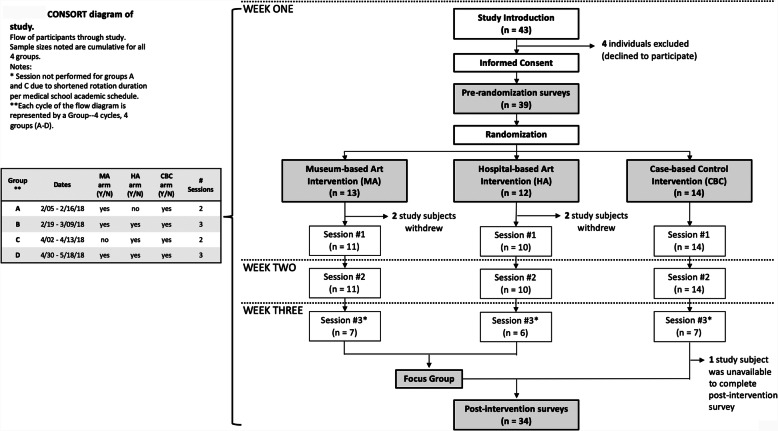
CONSORT diagram of study. Flow of participants through study. Sample sizes noted are cumulative for all 4 groups. Notes: * Session not performed for groups A and C due to shortened rotation duration per medical school academic schedule. **Each cycle of the flow diagram is represented by a Group--4 cycles, 4 groups (A-D)

The cluster-randomized, controlled pilot study using pre- and post-intervention assessments was conducted between February and May 2018 (Fig. 1). Eligible study participants included four groups of clinical medical students at the University of Michigan Medical School enrolled in the IM core clerkship at the Veterans Affairs Ann Arbor Healthcare System (hereafter “VA”). Two groups had been assigned a two-week clinical block (Fig. 1; Groups A and C) and the other two a three-week clinical block (Fig. 1; Groups B and D). All medical students rotate through the VA as part of the IM clerkship.

Study participants provided written informed consent. After providing informed consent, an independent statistician using a random number generator cluster-randomized each group of medical students in 1:1:1 manner to one of three interventions (described in depth below): Museum Arts, Hospital Arts, or hospital-based, Case-Based Control (“control”). Using this randomization scheme, all members of a single clinical group were exposed to the same intervention. Participants and study team were not blinded to group assignment and there was no crossover. Institutional Review Boards from both the VA and the University of Michigan Medical School deemed the study exempt. As this was not a clinical trial utilizing educational endpoints, it was not registered in National Clinical Trials registry. Full trial protocol is available upon requerst from the corresponding author. All study reporting was adherent with CONSORT guidelines [23].

### Interventions

Three interventions were utilized in this study: Museum Arts, Hospital Arts, or hospital-based, case-based control (“control”). Each study arm intended to include two to three weekly, one-hour afternoon sessions, for a total contact time of 3 hours per arm. Due to shortened clinical rotation, Session 3 was not completed for all participating students (Fig. 1). The timing of study activities was consistent across the arms and chosen to minimize impact on regularly scheduled clinical care and educational conferences. Groups received their interventions on the following dates: Group A, February 5 through February 16; Group B, February 19 through March 9; Group C, April 2 through April 13; and Group D, April 30 through March 18 (Fig. 1).

Museum Arts sessions were held at the University of Michigan Museum of Art (UMMA). Transportation was arranged to and from the museum. Sessions were facilitated by a trained academic art museum educator who had prior experience collaborating with medical professionals (RS). The one-hour sessions used visual art to explore themes of empathy, tolerance of ambiguity, mindfulness, and grit, as well as resilience and compassion. The Hospital Arts curriculum was identical to that of Museum Arts, except that a) it was held in a conference room at the VA, b) it was led by IM house officers (GS, SH, MT) with backgrounds in education and medical arts who were trained by the arts educator, and c) study participants interacted with printed and digital representations of the same works of art being discussed at UMMA (Supplemental Appendix A). The control arm consisted of case-based medical education sessions about common diseases encountered by IM physicians facilitated by the same house officers (GS, SH, MT) and were held in a conference room within the VA. (Supplemental Appendix B).

### Psychometric and educational outcome measures

Before randomization, participants completed paper-and-pencil surveys assessing demographic and arts-related variables, including voluntary participation in a Medical Arts program [24], prior art museum visits, formal or educational exposure to the visual arts, undergraduate study in the humanities, and intended specialty choice [25]. Baseline psychometric studies (Supplemental Appendix C) included the Jefferson Scale of Physician Empathy for Students (JSPE-S) [26], Tolerance of Ambiguity Scale (TOAS) [27, 28], Mindful Attention Awareness Scale (MAAS) [29], and Short Grit Scale (SGS) [30, 31]. These psychometric studies, our primary outcome measure, were repeated 2 days after the final intervention exposure due to study participants leaving the VA clinical site for clinical clerkships at other hospitals.

To evaluate whether incorporating the arts into the clerkship curriculum negatively impacts learners’ development of medical knowledge, we included an academic assessment as a secondary outcome measure. Post-intervention exam performance was assessed using the National Board of Medical Examiners (NBME) IM shelf exam, which was administered to all students at the conclusion of the clerkship. NBME shelf exams are standardized exams widely utilized in the United States to assess medical students’ acquisition of medical knowledge. These exams are administered at participating medical schools at the completion of core clerkships, including internal medicine.

NBME IM shelf exam performance allowed us to measure knowledge acquisition during the clerkship and assess if substituting art education for traditional didactic learning had an impact on academic achievement. If there were differences in NBME IM shelf exam performance between the groups, we could use each participant’s Medical College Admission Test (MCAT) national percentile to suggest whether differences in NBME IM shelf exam performance were due to differences in underlying test-taking abilities.

### Statistical analysis

We summarized the demographic characteristics and scores at baseline and post-intervention periods. We compared mean scores of JSPE-S, TOAS, MAAS, SGS, and educational outcome measures between arms of the study using analysis of variance (ANOVA). We compared the difference in differences between study arms from baseline and follow-up periods (i.e., to assess for whether there were differences between the three arms in the changes from pre-intervention to post-intervention) using repeated measures ANOVA. As this was a pilot study, we performed no formal power calculation; we utilized convenience sampling to arrive at sample size for the trial. Analysis was performed according to participants’ assigned groups and there was no crossover. No interim analysis was performed and the trial was not stopped early.

### Focus groups

Following completion of the post-intervention psychometric surveys, Museum Arts and Hospital Arts participants met for one-hour, semi-structured focus group discussions (Supplemental Appendix D) to gather participants’ perceptions and opinions of the intervention. We focused on groups receiving arts-based interventions. This was independent from any medical arts teaching. Focus groups were audio-recorded and were conducted by a female Masters’ degree-level research team member who was familiar with but did not have direct involvement in study interventions (SW) [32, 33]. Study participants were aware of the focus group facilitator’s role in the study. No other observers were present. Digital recordings were transcribed verbatim. All identifying information was removed from written transcripts prior to qualitative analysis. Focus groups were performed within COREQ guidance [34].

### Qualitative analysis

We conducted content analysis on focus group data using both deductive and inductive approaches. Two study team members with expertise in qualitative methods (SW, MQ), one of whom was blinded to the group’s exposure (MQ), reviewed a sample of focus group transcripts and created a preliminary coding scheme that included pre-identified deductive codes based on the study’s goals (e.g., participant reflections on art sessions, emotional impacts, and clinical applications) [35]. Additional inductive codes emerged from the transcripts. The same two study team members independently coded (line-by-line) the focus group transcripts. Coders discussed any discrepancies in coding until agreement and thematic saturation was reached. Data were aggregated, organized by code into table format, and synthesized to explicate findings. Study participants did not provide feedback on the findings. Qualitative analysis and data reporting were conducted under COREQ guidance [34].

## Results

### Study population

Participant flow is presented in Fig. 1. Forty-three students were approached; 39 provided consent and underwent randomization. Four individuals did not consent due to time commitment concerns. Of the 39 participants who consented, 35 completed all available interventions. One participant (control group) was unable to complete follow-up surveys, yielding data for 34 total participants. Due to the distribution of individuals who did not participate in the study, group A did not include a Hospital Arts arm and group C did not have a Museum Arts arm (Fig. 1).

Study arms did not differ significantly in terms of age, gender identity, race, undergraduate area of study, prior art museum exposure, prior attendance at a Medical Arts program event, or whether the decision of a future specialty had been made (Table 1). Among all groups, six (17.6%) participants had pursued non-natural science-based undergraduate degrees and one (2.9%) had pursued both non-natural science and natural science degree programs.

**Table 1:**
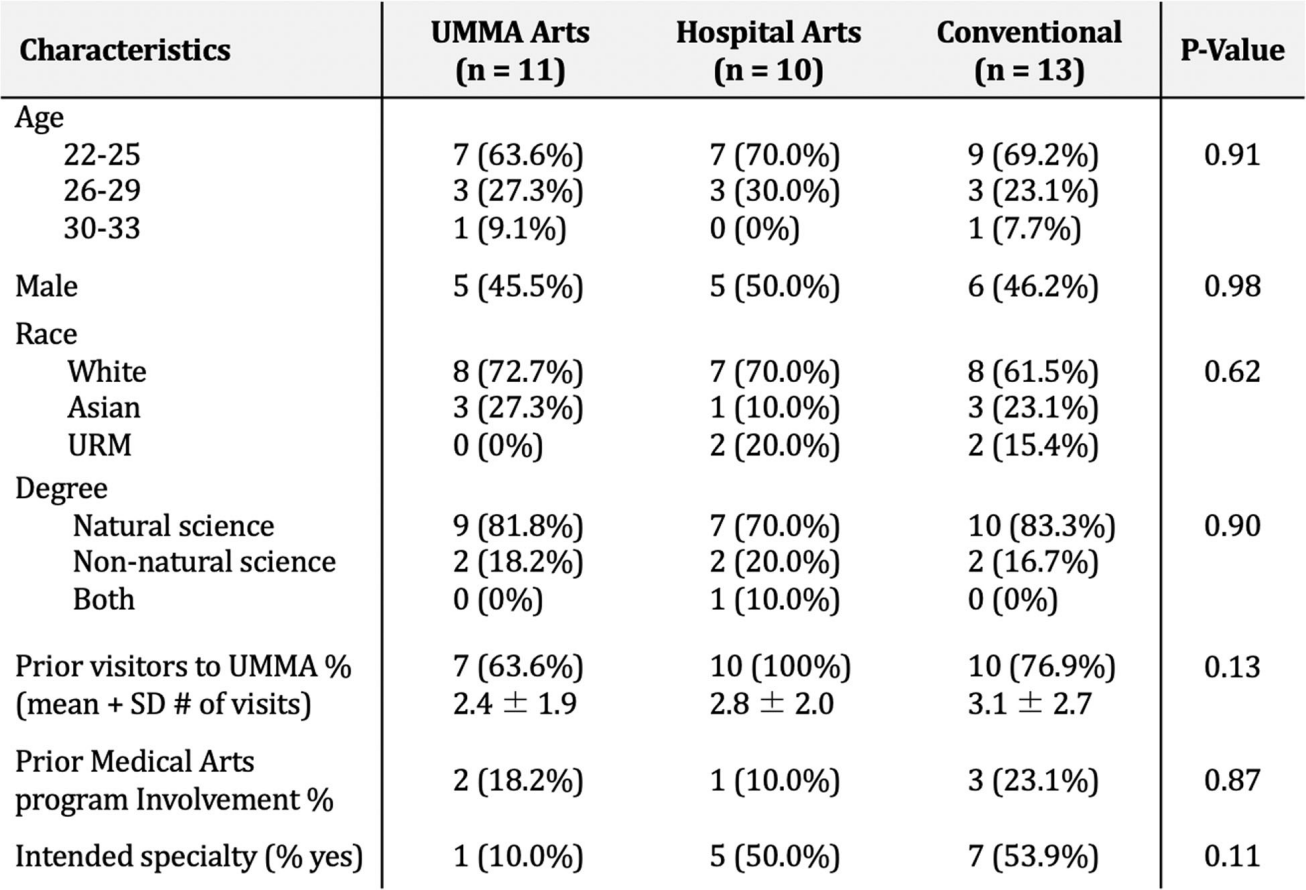
Summary of study participants

Baseline statistics of study participants by arm (with assessment of between-group statistical difference)

### Psychometric and educational outcomes

Comparison of baseline and post-intervention psychometric outcome surveys demonstrated no significant between-arm differences in JSPE-S, TOAS total score or sub-scores, MAAS, or SGS scores (Table 2, rows 1–8). Baseline measurements demonstrated no statistically significant between-arm differences in MCAT percentile (Table 2, row 9). Evaluation of NBME shelf exam performance after completion of the intervention revealed no between-groups differences (Museum Arts, 47.9 +/− 20.4; Hospital Arts, 66.6+/− 29.3; control, 7.5 +/− 22.8 percentile; *p* = 0.22 by ANOVA; Table 2, row 10).

**Table 2:**
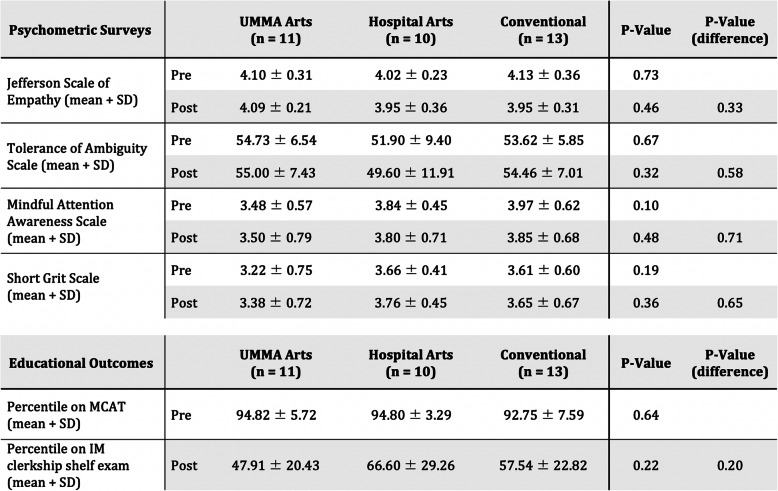
Quantitative outcome measures comparison

Pre- and post-intervention survey scores by arm. Comparison of between-arm statistical significance as well as pre- and post-intervention change in measures between arms

### Focus groups

Six focus groups organized by individual, arts-based intervention teams were conducted (participants: *n* = 11 Museum Arts, *n* = 10 Hospital Arts). Key themes are summarized below.

#### Participant reflections on the study

Study participants had largely positive responses to involvement.

*“I thought on the days when we didn’t have too much going on, it was a good break from kind of just sitting in the team room for most of the day. It was just a different way of thinking, it was a good mental exercise.”*

*“I really liked it, it was a nice break from the busyness of clinical rotations. It was something to look forward to.”*

*“I don’t know that it really changed … the way I kind of thought about things during my day to day work.”*

#### Clinical applications

Study participants thought the arts provided a space to practice empathy. They also thought the arts increased awareness and understanding of biases present in clinical care, especially in caring for patients with different backgrounds from themselves.

*“I think it’s helpful to be exposed to a wide range of things because our patients come from a wide range of backgrounds and it’s sometimes helpful to … figure out a way to connect with something that maybe you hadn’t necessarily connected with before.”*

*“I guess it … gave me some more insight into the biases that I take into certain interactions, especially in the hospital … to me that was really constructive to … not assume everybody’s going through the same type of experience.”*

Study participants randomized to arts interventions generally reported an improvement in their ability to differentiate between subjective and objective information, a focus of one intervention.

*“Something that I had trouble with before coming into this rotation was … separating out the subjective part of this whole process from the objective part and if you can … separate those two things … you can focus on the situation a lot better.”*

*“At one point, we were comparing and contrasting the difference between observing something objectively while also being able to experience it through the patient’s lens and sometimes those two things can be completely different and it was just … a nice way to think about things a little differently.”*

#### Emotional and psychological impact

Many study participants found the interventions to be calming and relaxing. They appreciated the opportunity to reflect, to think in a different way, to escape from a perceived scrutiny of medical knowledge, and to acknowledge their own limitations.

*“It was nice to … take a second and reflect on the entire experience that we’ve been going through because sometimes you’re so busy that you don’t really have a chance to think about it from a broader perspective … I think it’s helped … process this entire process of going through a rotation a little bit better.”*

*“It was nice to have a time when…the expectations on us weren’t to know things but rather to reflect on things…felt like a much lower stress environment.”*

*“Sometimes that’s not a realistic expectation of the part of who we are and being able to kind of recognize that…we have vulnerability and it’s just okay to have that.”*

*“I thought it was a good exercise in trying to get out of our own comfort zone and try to relate to people we may not know much about or encounter normally.”*

However, reactions were not universally positive. Participants stressed over leaving the clinical environment. They worried about time away from direct patient care, not least because they feared that their absence would have a negative impact on their clinical evaluations.

*“I think it’s just a little stressful for med students…being the bottom of the totem pole…you feel like you should stay.”*

*“It was stressful to take the time out of the day.”*

Conversely, participants stated the arts-based intervention reminded them of the importance of self-care and mindfulness.

*“I think it’s valuable in medicine to think about, like, taking care of yourself too because we focus on other people most of the day, so I think it’s always a nice reminder.”*

*“[Importance of] the meditation aspect, just taking a moment to reflect on how things are going, even if it’s an incredibly busy day, just making sure to take a step back.”*

#### Suggestions for improvement

Some study participants recommended making it possible for students to opt out of the arts-based experience, or incorporating it into a non-core clinical clerkship (although it should be noted that one of the study’s goals was to integrate the arts experience into a required part of clinical training).

## Discussion

This pilot cluster-randomized, controlled study demonstrated that incorporating a visual arts curriculum into an IM clerkship is, in fact, possible and that it can be done both in art museum and acute care hospital settings. We did not observe significant between-groups differences in measured psychometric outcomes, an unsurprising finding given the short intervention and small sample size. Within the limitations of our study, a complementary arts curriculum does not negatively impact standardized educational metrics, a finding that could ameliorate medical students’ concerns about lost time in the clinical environment.

The focus groups offered several conclusions that may not otherwise have been captured in a hypothesis-driven study. First, many participants reported that arts interventions offered a novel way of thinking and allowed for time to reflect on clinical experiences. They identified this time for reflection as a rare part of their education. Participants reported awareness of and changes in foundational clinical practice principles, such as developing increased empathic skills, recognizing personal implicit biases, and differentiating between subjective and objective information.

Second, while participants described the arts interventions as a welcome escape, many had a more negative response. Temporarily putting aside their clinical responsibilities led to feelings of stress and guilt. This effect seemed more pronounced for participants who physically left the hospital setting. It is possible, but unlikely, that the content of the arts interventions themselves contributed to this feeling of stress. It is more likely that the stress reflects the intense educational environment that characterizes medical school, one in which learners feel constant pressure to excel under the specter of constant evaluation [36]. In other words, by being physically removed from the hospital setting, students worried that their absence would reflect poorly on their clinical performance and subsequent evaluations, despite explicit assurances to the contrary by both medical educators and department administration. This stress of perceived absence may have negatively impacted participants’ experience on-site at the museum or distracted participants from the intended lessons of the experience. Therefore, it was encouraging that under these conditions, participants thought the intervention fostered mindfulness and a recognition of the importance of self-care in their medical career. That an arts-based curriculum could promote self-care was a promising conclusion from this pilot study and could inform both interventions and outcomes of interest in future studies.

These results are consistent with previous studies of short-term arts-based curricula for medical students [14, 15, 19]. In one study, participants completed three 90-min sessions led by art museum educators at a local museum. Similar to our population, participants reported improvement in their appreciation of varying perspectives, listening skills, and recognition of assumptions [15, 19]. In that study, students were self-selected — a key difference from our trial’s methodology. Our work suggests that arts-based curricula may be valuable for *all* medical students, not just those who choose to participate. Our data are also congruent with a study of surgical interns, which demonstrated that incorporating non-traditional educational efforts into the clinical space is personally and professionally beneficial [37].

Strengths of our study include use of a cluster-randomized design and outcome measures relevant to curricular stakeholders. Unlike some prior studies in the field, which struggled to garner faculty acceptance of incorporating less traditional concepts into medical training, we benefitted from strong institutional support for the central idea and purpose of the study [37]. The Hospital Arts arm of our study suggests that benefits of integrating arts into the curriculum may not be limited to institutions with ready access to an art museum or expert art museum educators. The museum environment is traditionally where the arts have been enfolded into medical education [38]. Trained arts educators can provide expert perspective and analysis, all the while enhancing an intervention as being “evaluation-free.” However, not all clinical training sites have access to art museums or trained arts educators. Armed with appropriate techniques, training, and curricula, medical educators may represent a potential solution.

It is unsurprising that we did not find between-groups differences in psychometric outcomes. This was a small, short duration trial, although for some individuals a single arts session may be sufficient to fundamentally change worldviews [38]. We chose this two- to three-week period at the VA during the medical students’ IM clerkship for both consistency and practical purposes. The VA IM clerkship is the only clerkship for medical students where all are simultaneously on the same rotation, have nearly equal clinical experiences, and are able to dedicate an additional hour weekly to the trial. Other hospital sites at University of Michigan provide varying internal medicine rotations (i.e., sub-specialty, outpatient internal medicine, and general inpatient internal medicine occur during the same time period). Moreover, our evaluations followed the interventions closely. After the passage of more time and the acquisition of more clinical experience, learners might look back on the arts experiences as being more valuable than they did immediately after the intervention.

More nuanced and robust conclusions will require larger studies, perhaps with longer duration of intervention, longer-term follow-up, or multiple sites. The nature of the arts stimuli may also have played a role in the observed outcomes. While prior studies of visual thinking strategies with similar duration of exposure have been reported as positive, these were not controlled, randomized, or tested in an exclusively clinical medical student population.

Logistical barriers included transportation, scheduling, and facility and personnel availability. As mentioned previously, many students were concerned that absences of any duration from the clinical setting would negatively impact their evaluations. One possible approach could be to move the intervention to a clerkship seen as less critical to students’ futures, or to make it elective. Yet, this self-selection would then defeat the underlying purpose of assessing whether incorporating the arts into the clinical space can be beneficial to *all* students.

## Conclusions

In this pilot trial, we demonstrate proof of principle for the integration of visual arts into the clinical educational space and for running a rigorous trial of the medical arts concept. Studies of arts curricula in medicine have not previously utilized randomization, contact time controls, nor have they focused upon the most at-risk subset of medical students. While there were no statistically significant changes in psychometric outcome measures, observation of and reflection on art did not jeopardize educational performance. Focus group discussions suggest numerous possible benefits of integrating an arts curriculum into the clinical space, as well as potential problems. Further coordinated, multi-institutional study is needed to more firmly establish the appropriate role of the arts in modern undergraduate medical education.

## Supplementary Information

**Additional file 1. **Strohbehn et al. – Arts in medicine R1 appendices. Supplemental appendices for *Visual arts in the clinical clerkship: a pilot cluster-randomized, controlled trial*. This file contains teaching scripts and works of art for the hospital arts and museum arts interventions (Appendix A), teaching scripts for the case-based control intervention (Appendix B), information regarding psychometric outcome measures (Appendix C), and guiding questions for focus group discussions (Appendix D).

## Data Availability

De-identified supporting data are available by written request and pending agreement by all authors. Focus group transcripts are redacted to ensure confidentiality. Any potentially identifying demographic information (name, date, age, race) will be removed from educational data to ensure compliance with the Family Educational Rights and Privacy Act (U.S. Code Title 20).

## Abbreviations

IM: Internal Medicine
NBME: National Board of Medical Examiners
MCAT: Medical College Admissions Test
VA: Veterans Affairs
JSPE-S: Jefferson Scale of Professional Empathy-Student
TOAS: Tolerance of Ambiguity Scale
MAAS: Mindful Attention Awareness Scale
SGS: Short Grit Scale
UMMA: University of Michigan Museum of Art
ANOVA: Analysis of variance
