# Supplementary material for: Visual arts in the clinical clerkship: a pilot cluster-randomized, controlled trial
Source: BMC Med Educ. 2020 Nov 30;20:481. doi: 10.1186/s12909-020-02386-w (PMC7708096; doi:10.1186/s12909-020-02386-w)
Supplement: Supplementary file 1 — Additional file 1. Strohbehn et al. – Arts in medicine R1 appendices. Supplemental appendices for Visual arts in the clinical clerkship: a pilot cluster-randomized, controlled trial. This file contains teaching scripts and works of art for the hospital arts and museum arts interventions (Appendix A), teaching scripts for the case-based control intervention (Appendix B), information regarding psychometric outcome measures (Appendix C), and guiding questions for focus group discussions (Appendix D). [file 12909_2020_2386_MOESM1_ESM.docx]

**Supplemental Methods Appendices for:**

*Visual arts in the clinical clerkship: a pilot cluster-randomized, controlled trial*

**Authors**

Garth W. Strohbehn, MD, MPhil^1,2,*^, Stephanie J.K. Hoffman, MD^1,3^, Molly Tokaz, MD^1^, Nathan Houchens, MD^1,2,3,4^, Ruth Slavin, MA^5,6,7^, Suzanne Winter, MS^3,4^, Martha Quinn, MPH^4,8^ David Ratz, MS^4,9^, Sanjay Saint, MD, MPH^2,3,4,9^, Vineet Chopra, MD, MSc^2,3,4,9^, and Joel D. Howell, MD, PhD^7,10,11,12,13^

1. Internal Medicine Residency Program, University of Michigan Medical School
2. Medicine Service, Veterans Affairs Ann Arbor Healthcare System
3. Division of Hospital Medicine, University of Michigan Medical School
4. Patient Safety Enhancement Program, University of Michigan and Veterans Affairs Ann Arbor Healthcare System
5. University of Michigan Museum of Art
6. Office of the Provost, University of Michigan
7. Medical Arts Program, University of Michigan Medical School
8. University of Michigan School of Public Health
9. Center for Clinical Management Research, Veterans Affairs Ann Arbor Healthcare System
10. Division of General Internal Medicine, University of Michigan Medical School
11. Department of Health Management and Policy, University of Michigan
12. Department of History, University of Michigan
13. Center for Bioethics and Social Sciences in Medicine, University of Michigan

all in Ann Arbor, MI, USA

***Current Affiliation:** Section of Hematology/Oncology, University of Chicago, Chicago, IL

**Corresponding author:**
Joel D. Howell, MD, PhD
Division of General Internal Medicine

North Campus Research Complex

2800 Plymouth Road, Building 16

Ann Arbor, MI 48109

T: 734-615-8342; E: jhowell@umich.edu

**Word Count:** 2972 (inclusive of headings)

**Appendix A**

**Museum-Based Arts-Based Education Intervention**

All Arts-Based Education Interventions were conceived of and developed by Ruth Slavin, MA, of the University of Michigan Museum of Art. These represent lessons developed utilizing artwork available only at the University of Michigan Museum of Art.

**Session 1: Compassion and Grit – by Ruth Slavin, MA**

Art: ***Death of Leander, Giulio Carpioni*** (oil painting, 1655)

***Vishnu as Varaha, the Cosmic Boar*** *(*Sandstone sculpture, 10^th^ century)

Activity:

Exploration of painting: *Death of Leander*

Guide group in close looking at scene, and elicit their ideas about both action and figure portrayal.

Invite them to share associations to past experiences and stories.

Connections to clinical experience (care, loss, death, roles)

Tell story of Hero and Leander, describe aspects of human loss embodied in this mythological painting. The actions of the gods, but the experience of the humans is foregrounded.

Additional discussion questions as needed:

*Describe the figures. Consider why the artist has included each figure and how they are depicted. You may want to consider age, gender, position, expressions and gestures, garments or associated objects.*

*Can you find any objects depicted that might be symbols?  What meanings do you associate with these objects? Based on your observations: what experience or ideas about human mortality is this work intending to communicate?*

*What message does the painting convey to us about the human experience of life and death?*

**
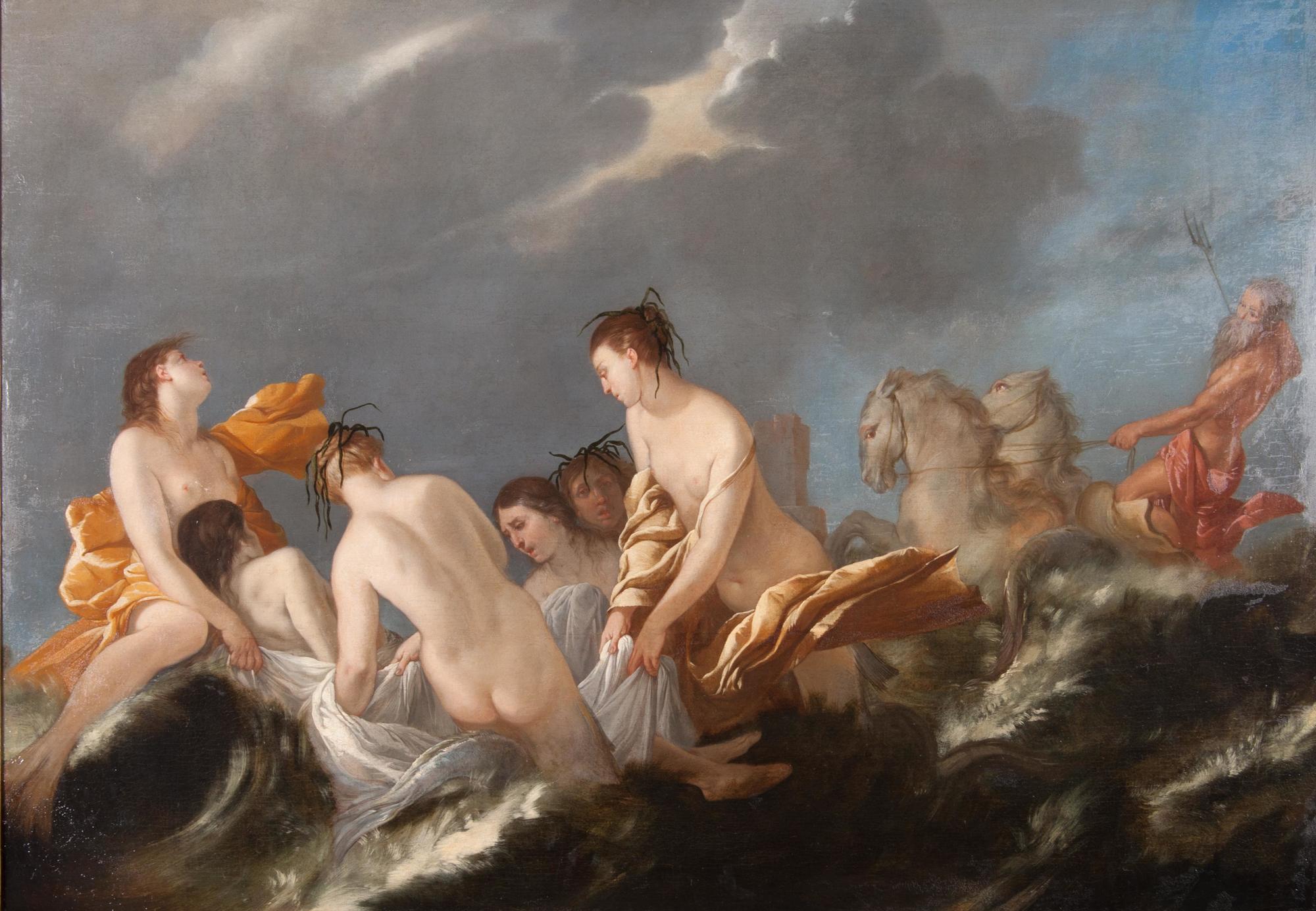
**

***Hero and Leander***

This painting depicts the drowning of Leander, a young man who was the lover of a priestess of Aphrodite named Hero. Every night Leander would swim across the Hellespont, the strait connecting the Sea of Marmara to the Aegean Sea, to the tower where Hero lived so as to pass the hours with his beloved. A winter storm, evoked in the painting by the turbulent waters and stormy sky, caught Leander during one of his nightly crossings, and he drowned. Four sea nymphs, accompanied by a merman and Poseidon in his sea chariot, have risen from the dark waves to support Leander's body in a white shroud. Their mourning and anguish over the dead lover foreshadows the impending sorrow and suicide of Hero, who still waits on her tower visible in the distance.

Guided looking at this artwork, exploring aspects of the work, subject matter and original location: like stained glass told important religious stories in images on the exterior of a temple in 10^th^ century. Hindu diety Vishnu, sometimes known as the Sustainer, comes to earth in a variety of guises, known as avatars.

***
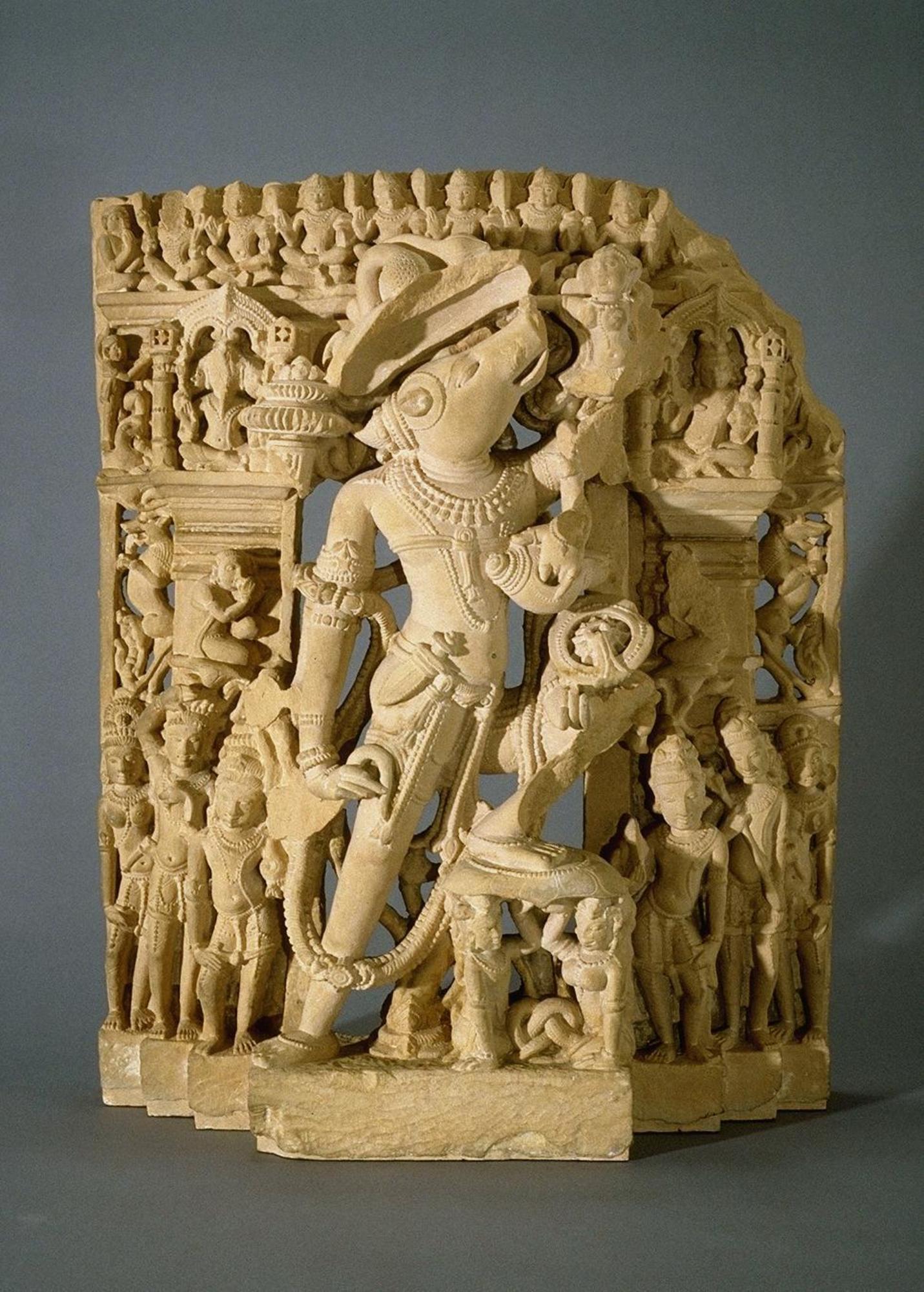
***

***Vishnu as Varaha, the Cosmic Boar***

The particular form is determined by the needs of the earth and it’s people—therefore Vishnu shows strategic understanding in his choice as well as empathy.

(Elicit student’s definition of compassion)

As physicians in training, your day may comprise many different situations each with their own demands. As caregivers, you may be fulfilling a leadership role but also bending and shaping yourself to what is needed. How to do relate to this concept of Vishnu and being expected to bend and mold yourself to fill the needs of those around you? How does that affect you (i.e., stress, burnout, depersonalization?)

Invite thoughts and responses.

Additional discussion questions:
*(Elicit student’s definition of compassion)*

*As physicians in training, your day may comprise many different situations each with their own demands. As caregivers, you may be fulfilling a leadership role but also bending and shaping yourself to what is needed.*

Introduce the issue of self-care for caregivers, and the prevalence of stress and burnout for the physician. Introduce a non-religious, non-denominational form of the loving kindness meditation, which can be modified to address many aspects of their experience: impact of loss and death, dealing with difficult people, and caring for the self.

*Invite thoughts and responses*.

Introduce the issue of self-care for caregivers, and the prevalence of stress and burnout for the physician. Introduce a non-religious, non-denominational form of the loving kindness meditation, which can be modified to address many aspects of their experience: impact of loss and death, dealing with difficult people, and caring for the self.

**Session 2: Empathy – by Ruth Slavin, MA**

**Art:**

***Marion Jones, Legs, Raleigh, North Carolina,***

Rick Chapman, 2001

***Sharecroppers Child (Abbreviated title)***

Dorthea Lange, 1937 creation date, reprinted in 1990 (The Library of Congress)

***Bethesda Fountain, Central Park***

Joel Meyerowitz, 1968

***Merce Cunningham****,* Annie Liebowitz, 1994

Activity

In this activity, you are invited to use your imagination to inhabit another person.

Choose a work of art that you are drawn to and write about your day in the first person (as if you were this person). If there is more than one person in the scene, identify one person to write about.

Pair up with a partner—who picked a different photograph.

Don’t read your initial writing yet, but point out the works you wrote about to each other.

Using close looking and observation, now write about your partner’s chosen photograph. This time, your goal is to describe what you can observe about this person from the outside, in a more objective or empirical description. Be as concrete and factual as you can in creating this second piece of writing.

Working with one photograph at a time, take turns sharing your writing about this person, working with one work (sharing both pieces of writing), then the other.

Return to the big group, inviting a few people to share one or both of their works and both pieces of writing. (2 works, 4 pieces of writing).


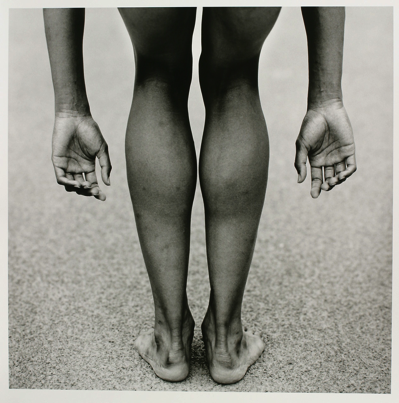

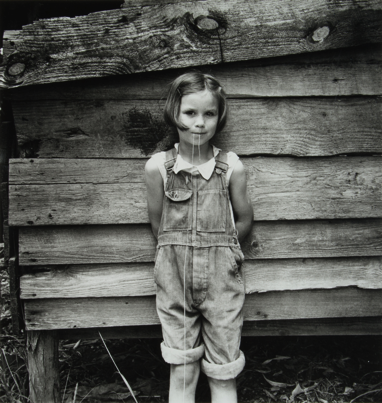

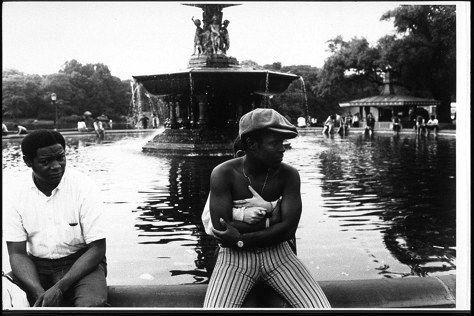

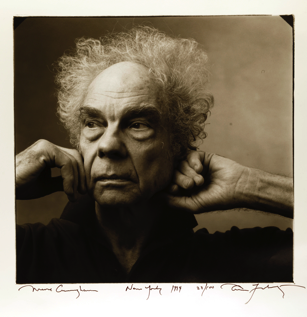


*Initial discussion questions:*

*What are the similarities and differences in the two descriptions? Is there common ground?*

*How was the* ***experience*** *of writing the second narrative different from the first?*

*How was the* ***tone*** *different?*

*Additional discussion questions:*

*How do you think the experience of "putting yourself in someone's shoes" is different from observing them?*

*What relevance do you see in these two approaches in the clinical setting?*

*What can the attempt to understand a person's subjective experience add to your experience and effectiveness (or your practice/effectiveness/creating good communication?*

*Optional extension of this activity:*

*Return to working with your partner: choose one of your “observation” writing pieces and work together. Explore* ***primary observations*** *such as the girl is frowning vs.* ***interpretation or secondary observation****: she is angry.*

*How much of the observation writing piece was observational vs. interpretation?*

*What stereotypes were you aware of that you avoided? What stereotypes did you play into?*

**Session 3: Resilience and Tolerance of Ambiguity – by Ruth Slavin, MA**

**Art:** Three art works by Felix Gonzales Torres:

***Untitled ( Dad), 1991*** *(Candy piece)*

***Untitled (Ross), 1991*** *(Candy Piece)*

***Untitled (March 5^th^) #2*** *(Light bulb piece)*

**Activity:**

*Today we will explore 20^th^ century works of art by American artist Felix Gonzalez-Torres.*

*Torres was born in Cuba, raised in Puerto Rico, and lived in New York City and Los Angeles. Gonzalez-Torres made works of art with ordinary objects—in this case light bulbs and extension cords/candy--the works themselves are a set of instructions, which give a lot of choice to the exhibitor or owner of the work****.***


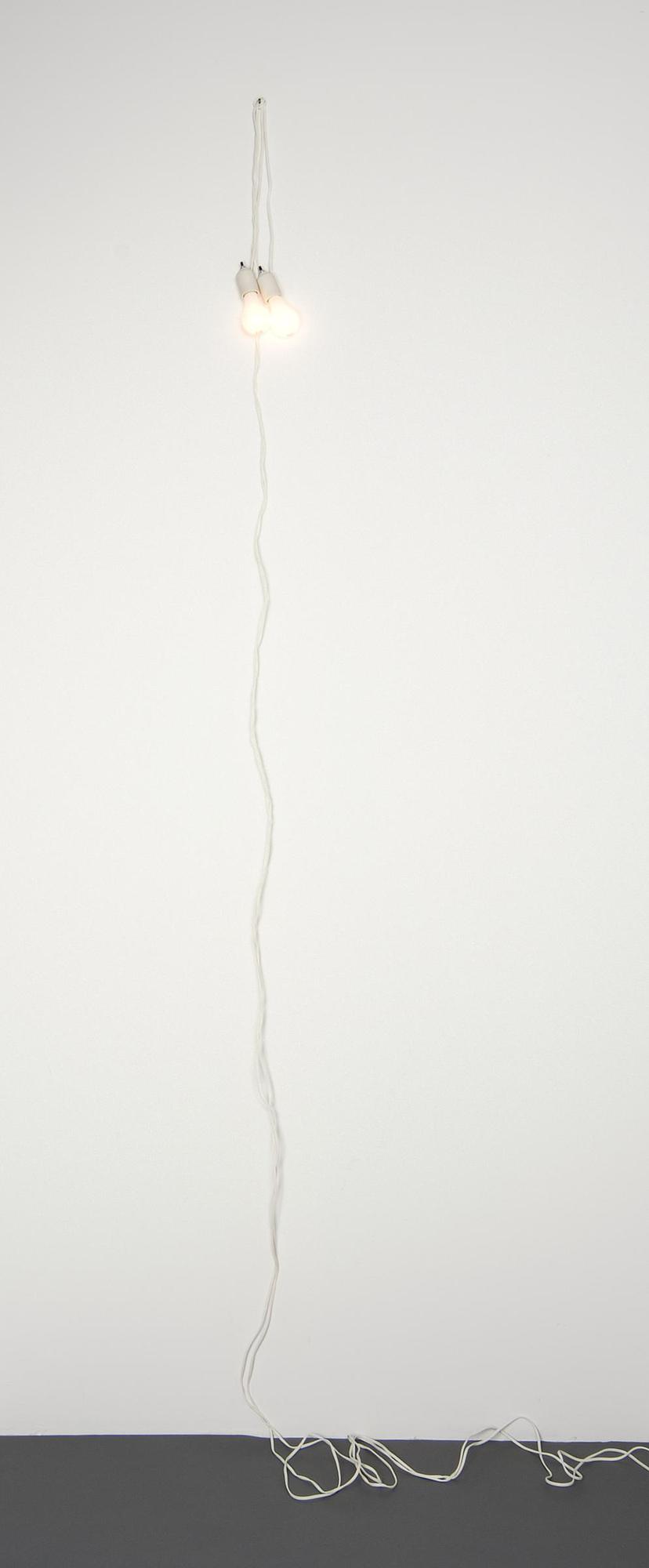


Take a few minutes to look at this object.

*Discuss your initial reactions. Do you think the objects or their arrangement are intended to be meaningful or symbolic?*

*What ideas, experiences, stories or questions come to mind as you look at this work of art?*

**Introduce participants to Gonzales Torres’ “Candy pieces”:**


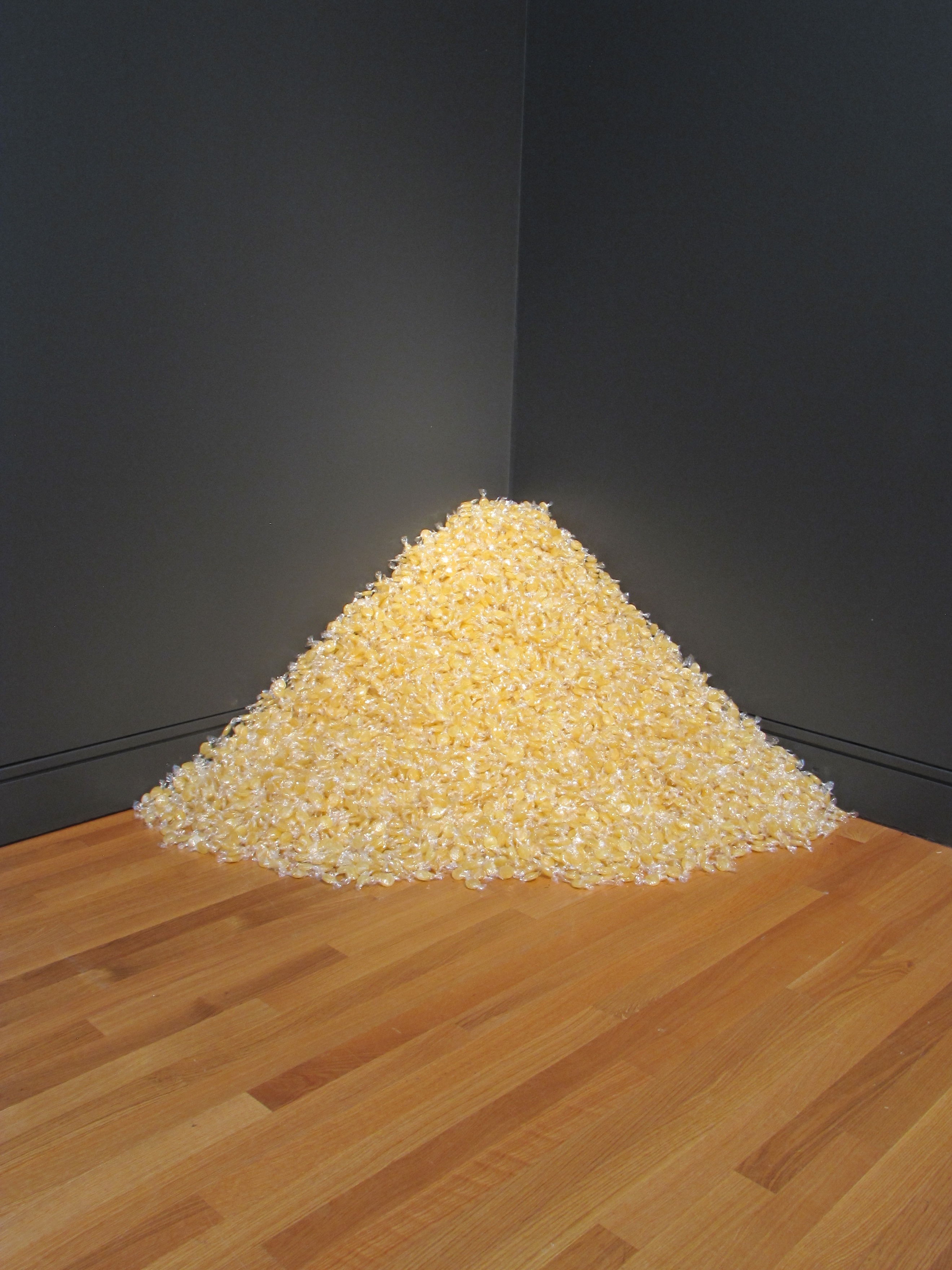

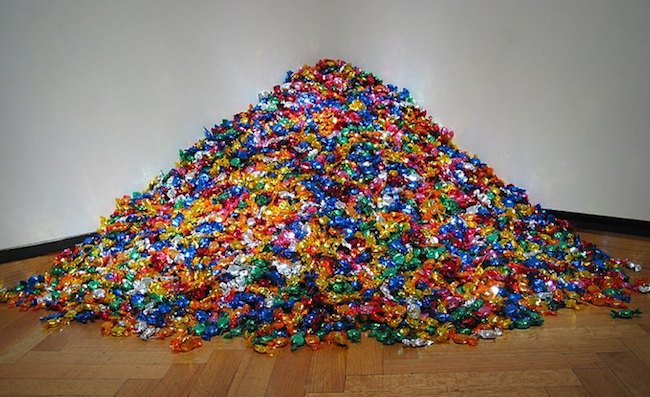


One of the interesting things about Gonzales Torres’ works that we are looking at is that they come with instructions—and to some extent the instructions ARE the work of art. In the case of the light bulb piece—eventually whether together or separately, the light bulbs burn out. It is up to the museum if and when to replace these. In the case of the candy pieces, the museum or owner arranges the candy as the museum decides. These two candy pieces are always to be shown with signage inviting people to take a piece of candy: the sign says “Please take only one.” After hearing these stories and the artist’s intention behind the work, how does this affect the way you see/experience/interpret/interact with the pieces?

Discussion questions:

*Discuss your initial reactions. Do you think the objects or their arrangement are intended to be meaningful or symbolic?*

*What ideas, experiences, stories or questions come to mind as you look at these works of art?*

*Why do you think the artists wanted the visitors to be invited to take a piece of candy?*

*How does this affect your thinking about the artist and the artworks?*

**Felix Gonzalez-Torres made 19 of these candy pieces works—all called *Untitled.***

**However, six of them had the name of a specific person in parenthesis and the original installation weight of the candy represented what Gonzales referred to as the “ideal body weight” of the person named. Sometimes he referred to this weight as the “healthy body weight of the average male.”**

*How is the depiction meaningful/powerful in light of this new information on artist intention?*

*What message does the painting convey to us about life and death?*

**This work is called Untitled (Dad), 1991 (L) and a second candy piece, *Untitled (Ross in LA)*, 1991. Ross Laycock was the artist’s partner—he died of complications related to AIDS in 1991.**

**Art Identification**

**All works are from University of Michigan Museum of Art Collections except where indicated.**

**Intervention 1**

***Death of Leander,*** Giulio Carpioni (oil painting on canvas, 1655). Museum purchase.

***Vishnu as Varaha, the Cosmic Boar*** *(*Sandstone sculpture, circa 10^th^ century), Artist unrecorded. Museum purchase made possible by the Margaret Watson Parker Collection Fund.

**Intervention 2**

Three art works by Felix Gonzales Torres:

***Untitled ( Dad), 1991*** (Candy piece). Gold wrapped candy, replenish as desired. Collection of Carlos and Rosa de la Cruz (private collection).

***Untitled (Ross), 1991*** (Candy Piece). Jewel colored wrapped candy, constant supply. Dimensions vary with installation, ideal weight 175 pounds. Art Institute of Chicago, promised gift of Donna and Howard Stone.

***Untitled (March 5^th^) #2*** *(Light bulb piece).* (40-watt light bulbs, extension cords, porcelain light sockets. [Museum purchase made possible by the W. Hawkins Ferry Fund](https://exchange.umma.umich.edu/quick_search/query?utf8=true&q=credit_line:%22Museum%20purchase%20made%20possible%20by%20the%20W.%20Hawkins%20Ferry%20Fund%22).

**Intervention 3**

Left top: ***Marion Jones, Legs, Raleigh, North Carolina*.** (Gelatin silver print on paper). Rick Chapman. 2001, printed 2002.

Right top: ***Sharecroppers Child (Abbreviated title).*** (Toned Gelatin silver print on paper). Dorthea Lange. 1937, printed in 1990: The Library of Congress. Gift from the Collection of David S. Rosen M.D.

Bottom left: ***Bethesda Fountain, Central Park.*** (Gelatin silver print on paper). Joel Meyerowitz. 1968. Gift of Selma & Gerald Lotenberg.

Bottom Right: ***Merce Cunningham.*** (Gelatin silver print on paper). Annie Liebovitz. 1994. Gift of Gay Delanghe.

**Appendix B**

**Case-Based Conventional Education Intervention**

**Session 1: Case-based Discussion**

ID: 56M

Chief Complaint: “My chest hurts”

History of Present Illness:

330AM: Awoken from sleep with a pain that began in abdomen and radiated into chest, through back, down both arms. Described numbness and tingling in the arms. Describes pain as 5/10 with a “ripping” sensation. Lasted a minute or so and resolved on its own without issue. 430AM: Pain came back and has been constant since then. He took an ibuprofen and rested, without his pain going away. Called ambulance and was brought in.

530AM: Arrives at AAVA ED.

Denies diaphoresis, shortness of breath. Does endorse nonproductive cough and is still coughing (similar to his smoker’s cough from the past, has not smoked since 2012).

***Prompts***

*Ask them if there are additional components of history they want to know*

***Prompts***

*BEGIN BUILDING THE DIFFERENTIAL*

PAST MEDICAL HX

COPD 01/2017 FEV1 2.22 (63) FVC 3.50 (76) ratio 63

Smoked until 2012

Previous EtOH abuse

FAMILY HX

DMII COPD (mom)

SOCIAL HX

Marital Status: Never Married

Other Information:

Work status: on disability

Marrital status: never, no kids

living with friend, doesn't know how long it will last

Service hx: PERIOD OF SERVICE - POST-VIETNAM

Medications

Albuterol 90mcg (cfc-f) 200d oral inhl Inhale 2 puffs PO INH Q4H PRN

Budesonide 80/formoter 4.5mcg 120d inh Inhale 2 puffs PO INH BID

Ipratropium bromide 17mcg 200d oral inhl Inhale 2 puffs INH

Pravastatin na 40mg tab Take one-half tablet PO QHS

(does not take baby ASA daily)

PHYSICAL EXAM

Vitals: BP 136/86 HR 67 RR 12 temp 36.8? temporal pulse ox 100% room air

*(Did not get BPs on both arms, in case the students ask)*

Constitutional: Patient is a 56-year-old male who is alert and oriented.

Answering questions appropriately. In no acute distress.

HEENT:  Head is normal cephalic atraumatic. No significant rhinorrhea. No

erythema or exudate in the posterior pharynx. Neck supple. Trachea is

midline.

Respiratory: Mild wheezing throughout. Slight prolonged expiratory phase. No

use of accessory muscles. No reproducible chest wall tenderness.

Cardiovascular:  Regular rate and rhythm.  Good perfusion to distal

extremities.

Abdomen: Soft. Mild midepigastric discomfort. Tympany to percussion. No

masses or herniations. No CVA tenderness.

Musculoskeletal:  No gross deformities.  No signifcant pedal edema.  Strength

is equal and symmetrical.

Neurologic:  Mentation as noted above.  No ataxia.

***Prompts***

ADDITIONAL PHYSICAL EXAM MANEUVERS?

ADD TO THE DIFFERENTIAL, develop pre-test likelihoods before sending labs or ordering imaging

Studies and labs

EKG (first slide, since it’s most likely that you’ll have this information first)

Sinus tachycardia, no evidence of ischemia, QT is a little long, QTc not bad

***Prompts***

**At this time building on the differential**

**Prompt them on imaging:**

Agree with them that CXR should have been obtained – but ask why they want one. Are there items on the differential that will be diagnosable on CXR?

Now the Imaging Question Comes Up: Introduce concept of ‘protocolling’ a CT and how every test answers a question you are asking. Not all CTs created equal, not all contrast boluses created equal. Do you need CT-PE? Do you need CT aorta? Do you need CTA? Something else?

Case Evolution

Out of concern for dissection, patient sent for DISSECTION PROTOCOL CT (regardless of what you’d seen on the CXR you would end up doing this anyway).

Patient was comfortable in appearance when he arrived. He did go to CT for

dissection protocol. He was awake, oriented, normotensive.

(Advance to “From Bad to Worse” slide)

When in CT, he started feeling bad. He stated he felt like he was going to pass

out. Transported back to the ED. Goes into Vfib arrest.

***Prompts***

***Introduce ACLS and the FIRST step in this case – SHOCK the patient.***

***Introduce the Hs and Ts:*** *Hypovolemia, hypoxia, hydrogen ion, hyper/hypokalemia/hypothermia; toxins, tamponade, tension pneumo, trauma, thrombosis (pulmonary AND cardiac)*

Case Evolution

Nearly 20 minutes of CPR until ROSC.

EKG obtained as part of standard post-code care (advance slide to show EKG, advance again to show comparison).

New EKG showing gigantic STEMI.

Taken to the cath lab (minor VA miracle) where we see the following. (advance slide)

***Prompt***

***How does it look?***

*(Advance slide) Show them the video played against the normal anatomy.* ***Ask what is missing****.*

*ENTIRE LAD.*

*Cath Result*

*Left Main                 0 has no angiographic stenosis.*

*LAD (overall)           100 has a proximal 100% occlusion with TIMI 0 flow.*

*An intervention was performed on this lesion.*

*The first diagonal has a 60-70% stenosis.*

*CIRCUMFLEX (overall)     20 has a proximal 20% stenosis. OM1 has a 20%*

*stenosis. LPL is small and has no angiographic*

*stenosis.*

*RCA (overall)            50 is a dominant vessel and has a proximal 40%,*

*mid 50% and distal 40% stenosis. The RPL and*

*RPDA have no angiographic stenosis.*

*(Advance slide) Thrombus is sucked out, wire advanced into LAD and balloon opened, pushing contrast we see reconstitution of flow.*

Case Evolution

Transferred to ICU and ultimately to the floor.

**Prompt**

**Checklist of medications** prior to discharge (ASA/gp2b3a, BB, ACE, statin, +/- diuretic if heart failure)

Post-MI TTE with persistently low EF, discharged with a lifevest

Case Evolution

All meds started and discharged to home, miraculously enough.

**Prompt**

**Did we do anything wrong here? What did we miss? Would we do anything differently?**

Remember the kinetics of troponin elevation – we only begin to detect them 2-4 hours after the onset of ACS.

Have to know the question we are asking of a lab test – and its limitations!

This will begin to change with high sensitivity troponin – soon at UH we’ll have the ability to rule out AMI with a single lab test or even with a couple measurements over a two to four hour period of time.

Take home points

1. CP DDX
2. ACLS review Hs/Ts
3. Brief cath anatomy
4. Checklist of meds
5. Timecourse of troponin elevation

**Session 2: Case-based discussion**

ID: 56M

Chief Complaint: shortness of breath

History of Present Illness: in the powerpoint

***Prompts***

***What qualifies something as an asthma exacerbation?***

***Ask them if there are additional components of history they want to know…***

Assume ROS negative

But ask them everytime they ask a question *why* they want that info

Display the pre-clinic spirometry, and work them through it.. quality control, pattern of the loop, and the numbers

***Prompts***

***BEGIN BUILDING THE DIFFERENTIAL FOR OBSTRUCTIVE LUNG DZ***

Back to PPT

The rest of the history is included in the PPT

***Prompts***

**Update the differential**

**Ask what else they want to know before they begin examining the patient**

**Ask what they’re going to be looking for on physical exam**

Back to PPT

Introduce the physical exam

***Prompts***

**ADDITIONAL PHYSICAL EXAM MANEUVERS? (ASSUME ALL NEGATIVE… INCLUDING PALPABLE CORD)**

**ADD TO THE DIFFERENTIAL, develop pre-test likelihoods before sending labs or ordering imaging**

LABS/STUDIES

Labs and CXR included in PPT

***Prompts***

**At this time building on the differential/updating the differential**

**Is anything moving up or down? (malignancy should move down b/c of clear CXR, maintained albumin, no weight loss)**

**Are there additional things you would want to know about this CBC? (answer: eosinophilia would be helpful if positive; assume the differential is normal)**

**Prompt them on additional labs/imaging:** What additional tests are needed?

REAL PFTs

**Prompt**

Have them build on the skills from the first go-round to walk you through the new ones

Interlude #1

Reversibility in COPD

Wildly different definitions depending on who you talk to

As stage gets worse, “reversibility” declines

But it’s not an independent predictor of survival

There are low rates of reversibility… but we still give lots of bronchodilators! Why?

Breaking into “reversible” patients and “nonreversible” patients

Reversible patients have benefit in FEV1 from BD

But so do nonreversible patients!

**Prompts**

Back to the case

Order of testing? What’s going to most drastically change your management? The answer doesn’t matter, just the buy-in.

Go through the slides:

HRCT – ask em what they see

Read: Negative, no bronchiectasis, no gross airway obstruction 2/2 tumor, no parenchymal damage, no lymphatic damage

A1AT – 126 (nl)

IgE – 1437 (super high!)

QTb neg

Allergen panel → timothy grass positive but o/w negative (can ask about whether they would order this knowing that HRCT was negative)

Weird add’l studies if they ask for them

ANCA neg

PR3 neg

MPO neg

**Prompts**

So how do we put this all together?

Features of classical asthma (IgE and an eosinophilic pattern with reversibility) and features of classical COPD…

Advance powerpoint to introduce the diagnostic idea of Asthma-COPD Overlap Syndrome

Interlude #2

ACOS!

Discuss the “typical” patients

**Prompts**

Therapeutic implications – ask them to think about how you treat asthma and how you treat COPD, chronically and in the acute setting

The difference is immunomodulation in chronic asthma and longer steroid tapers in exacerbations!

So making this diagnosis *does* matter

Back to slides

COPD – predominantly neutrophilic inflammation

Asthma – predominantly eosinophilic inflammation

At a certain point in the natural history they start to look at the same – and at the beginning of the end stages, once enough airway remodeling has occurred in asthma, they behave similarly

**Prompts**

So what should we do with this patient

Have we maximized his COPD therapy (anti-neutrophilic therapy)? In a way, yes.

Have we maximized his asthma therapy (anti-eosinophilic therapy)? NOPE

Introduce montelukast if they haven’t already

Introduce omalizumab (anti IgE)

Introduce benralizumab (anti IL5)

Back to slides

What we did

Started on montelukast - no more exacerbations!

Created a bigger plan for 2wk prednisone tapers if he recurs

IgE remains elevated but lower (~1100)

Skills we worked on

1. Obstructive lung dz differential diagnosis!
2. PFT reading
3. CXR interpretation
4. And then the advanced teaching points in the powerpoint

**Session 3: Case-based discussion**

ID: 68M

Chief Complaint: “I passed out”

History of Present Illness:

T-1wk: Black tarry stools. Thought it was related to his iron pills. No new NSAID use.

Earlier today: Passed out while watching TV. Denied prodrome. Happened in the morning. Found down by wife. Called EMS.

This has happened before. Three weeks ago he was moving a propane tank outside. Passed out w/o prodrome. Lost consciousness and not sure how long he was out. A week later he had another one when moving the same propane tank. Told his wife and went to OSH ED.

***They will probably want to know more about the workup at OSH****.*

*Push on the workup and causes of syncope*

*Break down into neurologic, cardiovascular, orthostatic causes*

Reveal some of that workup if they ask and tell them that the patient has AS in the PMH. If they do not ask for this information here, keep this as a surprise until a bit later in the case. You will go more slowly in connecting the AS with the GI bleed.

Denies fevers, chills, cough, diarrhea recently, chest pain, SOB, palpitations, headache, exertional problems.

***Prompts***

***Ask them if there are additional components of history they want to know…***

***Prompts***

***BEGIN BUILDING THE DIFFERENTIAL STRAIGHT AWAY***

PAST MEDICAL HX

Aortic stenosis s/p surgical aortic valve replacement in 2010

Atrial fibrillation on warfarin

Prostate cancer s/p prostatectomy

HTN

HLD

COPD with FEV1 47% on 2L home O2

FAMILY HX

CAD in both mother and father

SOCIAL HX

Lives in Cadillac with wife

Active smoker, 100PY history

4-5 drinks per week

Medications

Norco TID PRN

Gabapentin qhs PRN

Warfarin qhs

Digoxin qday

Atorvastatin qhs

Lasix qday

Metformin bid

Iron sulfate bid

Omeprazole daily

Allergies

NKDA

**Prompts**

Let’s update the differential at this point and flesh out these categories

PHYSICAL EXAM

Vitals: BP 110/60 HR 60 RR 20 temp 36.9? temporal pulse ox 94% room air

*(Did not orthostatics in case they ask… but if they do ask, double back and ask why)*

Constitutional: Patient is appears stated age, sitting up in bed. Answering questions appropriately. In no acute distress.

HEENT:  Head is normal cephalic. Has bruise over R frontal portion of head. No erythema or exudate in the posterior pharynx. Neck supple. Trachea is midline.

Cardiovascular:  Regular rate and irregularly irregular rhythm.  Good perfusion to distal

extremities. 4/6 SEM at RUSB with radiation to carotids

Respiratory: CTABL

Abdomen: Soft, nontender, nondistended, +BS

Extremities: 2+ edema to knees bilaterally, bruise evident on R thigh

***Prompts***

ADDITIONAL PHYSICAL EXAM MANEUVERS? (ASSUME ALL NEGATIVE… including diastolic component of the murmur)

If they don’t ask about the diastolic murmur, instruct them why and also inform of other PE maneuvers a/w regurgitation

Things we need to do for exam: Neuro, rectal, orthostatics, careful assessment of AI

**ADD TO THE DIFFERENTIAL, develop pre-test likelihoods before sending labs or ordering imaging**

STUDIES

EKG (first slide, since it’s most likely that you’ll have this information first)

Sinus tachycardia, no evidence of ischemia, QT is a little long, QTc not bad

***Prompts***

**Other studies?**

Can show the EKG

If they ask about head CT say that’s a great idea – strike a balance between too much testing and not enough testing. Choosing Wisely recommendation *against* head CT in asymptomatic adults with syncope who have insignificant (not *zero*) trauma and normal neuro evaluation – from the American College of Emergency Physicians and we’ve extended it to our inpatient population

**Other calculations?**

Reticulocyte production index – marker of turnover etc

**At this time building on the differential**

**Hospital Course**

Given Hgb drop…. Pursue EGD showing…

(Flash EGD images [negative])

(Show capsule endoscopy images [AVM])

**Thinking about mechanisms**

Association between AS and AVM → Heyde’s

Brief history of Heyde’s

Mechanism

**Prompt**

Is it because of *more* AVMs or is it because they’re more likely to bleed?

Is there a way to study this?

**Flash studies**

**Conclusion**

What happened? TAVR! No evidence of bleeding since. Continued on PLAVIX (antiplatelet therapy in post-TAVR patients)

**Appendix C**

**Description of Psychometric Outcome Measures**

Psychometric studies included the Jefferson Scale of Physician Empathy for Students (JSPE-S), Tolerance of Ambiguity Scale (TOAS), Mindful Attention Awareness Scale (MAAS), and Short Grit Scale (SGS). JSPE-S is an established 20-item inventory developed specifically for assessing empathy in medical students.^25^ Higher scores reflect behavioral tendency towards increased empathy in patient care. TOAS helps to measure the extent to which a person finds ambiguous situations threatening rather than desirable. Higher scores reflect a greater level of tolerance for ambiguity. This psychological construct has been incorporated into the medical student selection process as a desired characteristic in a student trainee and physician.^26^ MAAS was chosen for its previously validated focus on self-regulatory aspects of mindfulness.^28^ Higher scores reflect increased levels of mindfulness in day-to-day experiences. Grit is a psychological construct encompassing perseverance toward long-term goals despite adversity, failure, and plateaus. The SGS is an eight-item inventory that was chosen to assess students’ grit due to its brevity and prior use in undergraduate medical students.^29,30^

NB: References refer to those in the main manuscript.

**Appendix D**

**Focus group guided discussion template**

**Focus Group Discussion Guide**

Draft – Below are proposed questions. Not all questions will be asked. We will tailor the questions to the interviewee and use questions that worked well in prior focus group discussions.

**Audio Recording Consent Script**

Study staff- Please give each participant the study informational letter and ask him or her to review the document.

Prior to turning on the audio recorder, please provide the participants with the following verbal information.

“You have received an informational letter with additional knowledge about the study and your participation in the focus group discussion or interview. “

“Everything you say will remain confidential. You also have the right to leave at any time or not answer any questions. We will be recording the interview to ensure that we capture everything you have to say accurately. Before we turn the recorder on, do you have any questions for us?”

When all questions have been answered, while the audio-recorder is still off, please ask- “Do you give consent to having your voice recorded?”

If anyone answers “no”, they cannot be audio recorded.

Please then inform the participates, “When the recorder is turned on, I will ask again, Do you consent to having your voice recorded? And you will state, “Yes”, are there any questions?”

Turn the recorder on and repeat the above phase and proceed with the focus group discussion or interview.

Draft – Below are proposed questions. Not all questions will be asked. We will tailor the questions to the interviewee and use questions that worked well in prior focus group discussions.

**Introduction:**

- Introduce project team and describe what their role will be.
- Explain the purpose of the focus group.
- Guarantee confidentiality from the team and ask participants to also maintain confidentiality.
- Ask if anyone has any questions.

**Staff Notes- Art themes**

- Resilience
- Grit
- Compassion
- Empathy
- Tolerance of ambiguity

1. **Please tell us how you found the art education beneficial?**

- *Interviewer Probes*:
  1. Interesting?
  2. Calming - relaxing?
  3. Mind clearing?

1. **Please tell us how you found the art education not helpful for?**

- *Interviewer Probes*:
  1. Lack of detail?
  2. Time away from patient responsibilities?
  3. Not interested in art?
  4. Question relevance to clinical medicine

1. **Please tell us areas where we could improve this education?**

- *Interviewer Probes*:
  1. Too short - long?
  2. Transportation?
  3. Surveys?
  4. Add?
  5. Delete?
